# Biotransformation and Cytotoxicity Evaluation of Kraft Lignin Degraded by Ligninolytic *Serratia liquefaciens*

**DOI:** 10.3389/fmicb.2019.02364

**Published:** 2019-11-21

**Authors:** Anil Kumar Singh, Pooja Yadav, Ram Naresh Bharagava, Ganesh Dattatraya Saratale, Abhay Raj

**Affiliations:** ^1^Environmental Microbiology Laboratory, Environmental Toxicology Group, CSIR-Indian Institute of Toxicology Research, Lucknow, India; ^2^Academy of Scientific & Innovative Research, Ghaziabad, India; ^3^Department of Microbiology, Babasaheb Bhimrao Ambedkar University, Lucknow, India; ^4^Department of Food Science and Biotechnology, Dongguk University Seoul, Seoul, South Korea

**Keywords:** kraft lignin, *Serratia liquefaciens*, biodegradation, biotransformation, cytotoxicity

## Abstract

Various chemical compounds emerged including kraft lignin (KL) during the processes of papermaking. These chemical compounds in effluent of the paper industry have hazardous environmental impacts. KL is liable for causing pollution of aquatic and water bodies; hence, it must be minimized in order to maintain a healthy and sustainable environment. In the present study, KL degradation was performed with ligninolytic bacterium *Serratia liquefaciens* and we confirmed biotransformation of KL to various less polluted or harmless compounds. KL being degraded as 1000 mg/L^–1^ concentration with incubating 30°C for 72, 168, and 240 h, shaking at 120 rpm under laboratory conditions. We found 65% maximum degradation of KL and 62% decolorization by the treatment with *S. liquefaciens* for 240 h (10 days). After being the treatment of KL, clear changes were observed in its morphology (using scanning electron microscopy and stereo microscopy), hydrodynamic size (using dynamic light scattering), and the functional groups [using Attenuated Total Reflectance Fourier Transform Infrared Spectroscopy (ATR–FTIR)]. Biotransformation of KL monitored by Gas Chromatography–Mass Spectrometry (GC–MS) revealed formation of various metabolites. In addition to degradation of KL, detoxification (involving biotransformation into various metabolites) was assessed using cytotoxicity assays 3-(4,5-dimethylthiazol-2-yl)-2,5-diphenyltetrazolium bromide [MTT and calcein-acetoxymethyl (AM) assays] using a human kidney cell line (NRK-52E), which indicated improved cell survival rates (74% for the bacteria-treated KL solution treated for 240 h) compared to the control (27%). Thus, the present study suggests that bacteria *S. liquefa*ciens might be useful in reducing the pollution of KL by transforming it into various metabolites along with cytotoxicity reduction for environmental protection.

## Introduction

Paper manufacturing industries are one of the world’s most polluting industries, emit, and expels highly toxic compound into the environment (Ince et al., 2011). In the pulp and paper industry’s effluents, significant quantities of toxic chemicals have reported. These toxic chemicals in effluent generated during the different paper manufacturing process including kraft pulping, bleaching, delignification, etc. may be responsible for various serious environmental concerns. The high concentration of various chemical like sodium hydroxide, sodium sulfide, sodium carbonate, bisulfides along with chlorine elements (chlorine dioxide), hydrochloric acid are the chief ingredients of wastewater of paper industries (Suresh and Hung, 2004). Apart from enormous quantities of different harmful chemicals, lignin and various forms of lignin-derived compounds are responsible for the dark color of wastewater and contribute a high level of aquatic pollution when drained into it (Nath, 2016).

Kraft lignin (KL) is a major component of paper industries, which released through effluent during the delignification process. Many researchers have reported that KL is a key component of wastewater, causing color appearance and toxicity (Shi et al., 2013; Verma and Ekka, 2018). Similar to natural lignin, because the complicated three-dimensional structure of KL, a range of ether and carbon–carbon bond is extremely recalcitrant to chemical and biotransformation/biological degradation. KL colors even at a small level in water are noticeable (25 mg L^–1^), whereas paper industry effluent typically contains >200–600 mg L^–1^ KL (Singh, 2012; Raj et al., 2014; Haq et al., 2016). KL blocks the photosynthesis in the aquatic ecosystem and reduces the oxygen level, which often leads to toxicity in the aquatic ecosystem (Sponza, 2003; Latorre et al., 2007). Hence, the degradation of KL provides the biotransformation of KL in paper industry effluent during the degradation or treatment process.

Over the years, several lab-scale KL removal methods have been developed using physical and biological methods. Processes based on physical methods (sedimentation, coagulation, oxidation, etc.) and chemical techniques (adsorption, microfiltration, etc.) are effective but costly for large-scale application (Kamali and Khodaparast, 2015). Biological treatment fungal, bacterial, algae, and plant systems methods seem to be accessible to evaluated for the degradation or biotransformation of contaminants in paper industry effluent, including KL. More recently, biotransformation involving specific bacteria and fungi has been receiving more attention. During the last decade, several bacteria and fungi with the ability to degrade or biotransform various types of lignin have been isolated. The abilities of microorganisms to degrade lignin are due to their ligninolytic enzymatic systems, which include the following “ligninolytic”: lignin peroxidases (LiPs), laccases (LACs), and manganese peroxidases (MnPs) (Chandra and Chowdhary, 2015; Chowdhary et al., 2018). These enzymes were initially reported in fungi and subsequently in bacteria (Saratale et al., 2009; Bugg et al., 2011; Harms et al., 2011). It has been reported that the bacterial ligninolytic system is more effective than the fungal system in terms of degradation and biotransformation of various forms of lignin into various metabolites (El Hanafy et al., 2008). The excellent enzyme production capabilities of bacteria have led to the huge demand for bacterial isolation that produces potential ligninolytic enzymes for degradation of paper mill pollutants by transforming into less toxic compounds. Recently reported, potential ligninolytic bacteria like; *Bacillus*, *Arthrobacter*, *Nocardia*, *Pseudomonas*, *Streptomyces*, etc. by various investigators has shown their paper mill pollutants degradation potential (Chandra et al., 2007; Tian et al., 2016). Despite the key role-playing, ligninolytic bacteria have been focused on their enzymatic activity that’s positively correlated with lignin mineralization and degradation (David Boyle et al., 1992).

The bacterial ligninolytic enzyme system is considered more stable in term of catalysis mechanism in a harsh environment than the fungi system; bacteria can usually accept a wider variety of habitats and grow more quickly than fungi (Harms et al., 2011). Furthermore, as contrasted to ligninolytic fungal enzymes, bacterial ligninolytic enzymes may be highly active in high temperatures and much stable, in high pH values, and concentrations of strong chloride and other organic chemicals (Sharma et al., 2007; Bugg et al., 2011; Dwivedi et al., 2011). LiP is an oxidoreductase enzyme, extracted and purified from fungi, which further employs for remediation of toxic compounds and colored removal from paper mill effluents (Mehboob et al., 2011). Apart from fungi, bacterial LiP has great potential as a ligninolytic enzyme due to huge biochemical versatility and adaptability to the environment of bacteria. Recently, we reported a LiP producing bacterium *Serratia liquefaciens*, isolated from effluent contaminated soil by lignin enrichment method, was able to degrade and detoxify pulp and paper mill effluent with significant reduction of lignin, 58% (Haq et al., 2016). Since this isolate degraded significant amount of lignin, but its biodegradation process need to explore for greater understanding.

The present work was to assess the KL degradation ability of *S. liquefaciens* and characterization of its biodegradation process along with toxicological evaluation of the sample after bacterial treatment. This study has demonstrated KL biodegradation/biotransformation ability of this isolate, and has shown cytotoxicity reduction on human kidney cell lines. We found significant degradation, color removal, as well as cytotoxicity minimizing potential after employing *S. liquefaciens*.

## Materials and Methods

### Bacterial Inoculum Preparation

The ligninolytic *S. liquefaciens* strain was isolated from the effluent of the paper mill (Haq et al., 2016). The isolate kept and maintained at −20°C for long-term usage. It was used to inoculate mineral salt medium (MSM) agar plates (pH 7.6) containing the following media composition (g L^–1^): Na_2_HPO_4_, 2.4; K_2_HPO_4_, 2.0; NH_4_NO_3_, 0.1; MgSO_4_, 0.01; CaCl_2_, 0.01; KL (Cat. No. 471003; Sigma–Aldrich, St. Louis, MO, United States), 1.0; D-glucose, 10.0; peptone, 5.0; and agar powder, 15.0. After 120 h of growth at 30°C, the bacterial colonies were used to inoculate 50 mL MSM broth by incubated for 24 h in flasks in an incubator shaker at 30°C. This freshly prepared bacterial culture was used to conduct the concerned experiments.

### KL Degradation Assay

Kraft lignin biodegradation assay was conducted using KL (molecular weight, ∼10,000 Da), which was purchased from Sigma–Aldrich (Cat. No. 471003, St. Louis, MO, United States). It has low sulfonate content (4%) and water-soluble. The KL degradation assay was performed in a flask (1000 mL) containing 500 mL MSM and KL (1000 mg L^–1^, measured COD 4030 mg/L) adjusts pH at 7.6. MSM + KL-containing flask was inoculated with 1% (v/v) fresh bacterial culture suspension followed by incubated at 30 ± 0.5°C on an orbital shaker (Kuhner shaker) at 120 rpm. The samples were harvested at 0 h (before inoculation) and 72, 168, and 240 h after inoculation. The <24 h sample was used as the control or untreated. These conditions were used as the standard conditions for all KL degradation/biotransformation experiments (Ravi et al., 2019).

The harvested samples were assessed for bacterial growth, LiP activity, color, and lignin content. Optical density measurement was used to determine bacterial growth (OD) at 600 nm of the culture broth using a double-beam (UV) visible spectrophotometer (Shimadzu, Japan), with a non-inoculated medium as the blank control. LiP activity was measured by centrifugation of 2 ml MSM broth culture, and centrifuged at 10,000 rpm at 4°C for 10 min. Supernatants were assessed based on the OD at 310 nm following H_2_O_2_-consistent veratryl alcohol-dependent oxidation to veratraldehyde (Tien and Kirk, 1988). The activity of the enzyme was measured as IU/mL. The quantity of enzyme required to oxidize a micromole veratryl alcohol per milliliter per minute was recognized as a unit of LiP activity. The color intensity of the culture supernatants was assessed based on the OD at 465 nm using the Canadian Pulp and Paper Association (CPPA) (1974) method, after the pH of the supernatant has been set to 7.6. The estimation of lignin content was conducted using the Pearl and Benson (1940) method. Triplicate experiments were conducted and the values reported as mean ± *SD* (*n* = 3).

### Characterization of KL Degradation

The KL degradation assay conducted under standard conditions as described above. KL was extracted from the culture medium using the acid precipitation method (Chen et al., 2012; Lu et al., 2017). The dried KL powder used to characterize using scanning electron microscopy (SEM) and stereo microscopy.

### Scanning Electron Microscope and Stereomicroscope Analysis

Structural and surface changes during the KL degradation assay induced by *S. liquefaciens* have evaluated at various time intervals by SEM and stereomicroscope analysis. KL powder samples were used Mini Sputter Coater to coat with gold (Model SC7620, Quorum, Technologies, United Kingdom) and followed by examined using SEM (Quanta 450 FEG, FEI, Netherlands) and energy dispersive X-ray (EDX) spectroscopy analysis. The morphology of the KL powder was also observed using a stereomicroscope (KH-7700, HIRO, Japan).

### Dynamic Light Scattering Measurements

A dynamic light scattering (DLS) technique was used to quantifying diameter hydrodynamic (*D*_H_) of the KL particles. The KL samples were diluted as 1/10 in MiliQ water. Followed by mean *D*_H_ in the KL samples were analyzed using a Zetasizer Nano-ZSP system (Malvern Instruments Ltd., Malvern, United Kingdom).

### Attenuated Total Reflectance–Fourier Transform Infrared Spectroscopy Analysis

The Attenuated Total Reflectance (ATR)–Fourier Transform Infrared (FTIR) spectra of the KL powder samples (control and bacteria-treated) were recorded using a single-beam ATR–FTIR spectrometer (Nicolet^TM^ iS^TM^, Thermo Fisher Scientific, United States). ATR–FTIR equipment has configured for 16 scans in the range of 4000–500 cm^–1^ and a resolution of 4 cm^–1^.

### Gas Chromatography–Mass Spectrometry Analysis for Metabolite Identification

Treated KL samples with SL and control sample prepared with cell-free culture supernatants in 50 mL quantities. Samples were extracted and derived using ethyl acetate and *N*,*O*-*bis* (trimethylsilyl) trifluoroacetamide with trimethylsilyl chloride (BSTFA + TMCS), respectively. A Gas Chromatography–Mass Spectrometry (GC–MS) spectrometer used sample by injecting the silylated samples (1 μl) (Ultra TSQ Quantum XLS Mass Spectrometer, Thermo Fisher Scientific, United States) according to the standard procedure (He and Aga, 2019). By comparing detected mass spectra to the compounds library of National Institute of Standards and Technology (NIST) (available with the spectrometer) identified as compound or metabolites present in samples.

### Cytotoxicity Evaluation

Samples were determined to be cytotoxic by the used of 3-(4,5-dimethylthiazol-2-yl)-2,5-diphenyltetrazolium bromide (MTT) and calcein-acetoxymethyl (AM) assays, described as standard procedures by Singh et al. (2015). When this kidney cell line (NRK-52E) was used for both assays. The cells kept in Dulbecco’s modified Eagle’s medium (DMEM) supplemented with 10% heat-inactivated fetal bovine serum (FBS) and 1× antibacterial-antimycotic (ABAM) solution in a 5% CO_2_ atmosphere at 37°C. The concerned cell line was used after they reached the confluence of 75–80%. Quickly, 2 × 10^5^ cells were grown on 96-well plates, and maintained for 24 h in a CO_2_ incubator. The treatments involved incubation for 24 h at 37°C with 50 μl of the untreated and bacteria-treated (72, 168, and 240 h) KL samples and 200 μl of the above-mentioned medium.

For the MTT assay of cytotoxicity, after 24-h KL treatment, MTT (GIBCO-Life Technologies, United States) added L^–1^ to each 96-well plates well with a final concentration of 0.5 mg. Further incubation at 37°C was performed for 2–3 h. The medium was removed, and dimethyl sulfoxide (DMSO) formed the formazan crystals. The density of formazan crystals corresponds immediately to the sum of viable cells. The OD was measured at 570 nm with a Spectra Max MS Multimode Microplate Reader (Molecular Devices, United States).

For the AM assay of cytotoxicity, after 24-h KL treatment at 37°C, the medium of culture was removed and cells washed with 1× phosphate-buffered saline (PBS), and then the diluted calcein-AM (200 mL) was added at the final of a 2 mM. After incubation at 37°C for 30 min, cells were visualized and quantified under a fluorescent microscope at 10× magnification (Nikon Instruments Inc., United States) with an excitement rate of 488 nm and emission filters with 520 nm. The statistical analysis was performed using the statistical program GraphPad Prism (San Diego, CA, United States) (Swift, 1997). The results are reported as mean ± *SD* (*n* = 3).

## Results and Discussion

### KL Powder Characterization

**FIGURE 1:**
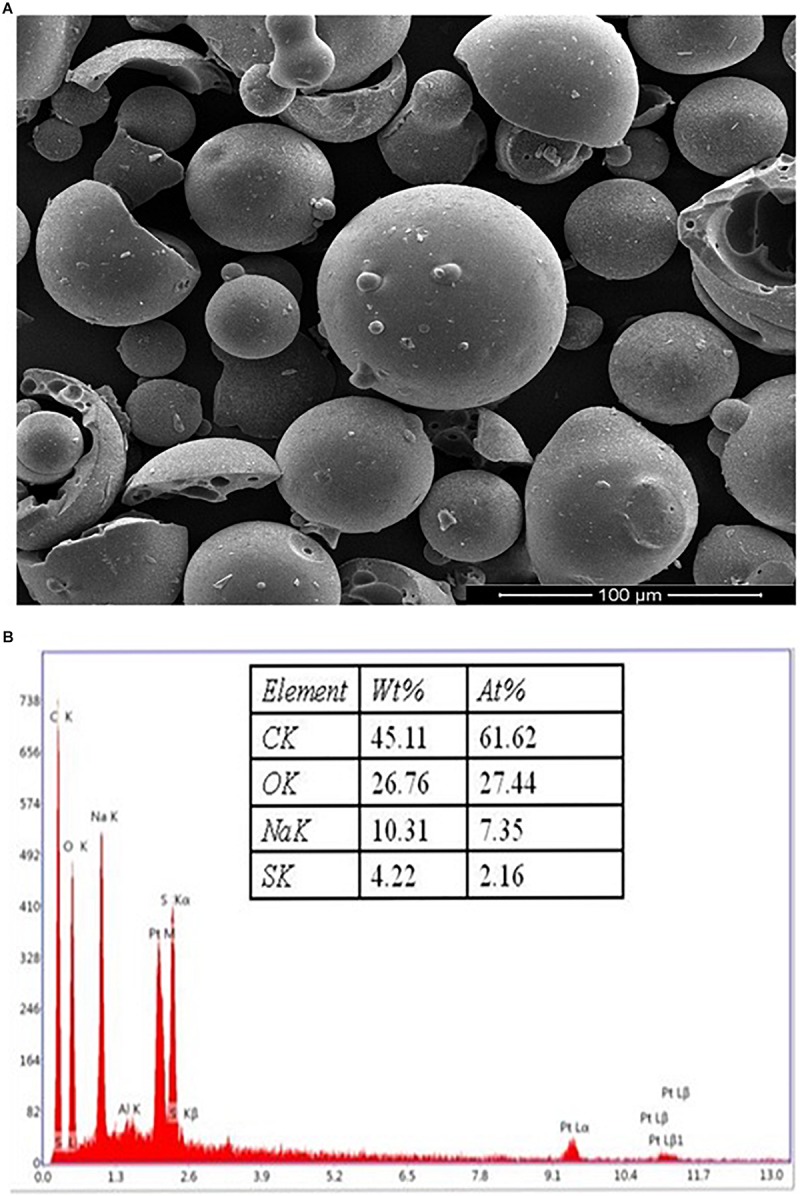
**(A)** The SEM images of the kraft lignin particles, showing rounded spherical shapes with smooth surfaces. **(B)** EDX spectra of KL powder.

The surface morphology of the KL powder (obtained from Sigma–Aldrich, St. Louis, MO, United States) was characterized by SEM and EDX analysis. The morphological observations might be by the KL extraction method from black liquor (Fierro et al., 2006; Pua et al., 2011). The SEM images of the KL particles show rounded spherical shapes with smooth surfaces as depicted in Figure 1A. The EDX spectroscopy elemental analysis revealed that the KL powder had a low sulfur content (4.22%) as shown in Figure 1B.

### KL Biodegradation Characterized by Spectrophotometric Analysis

**FIGURE 2:**
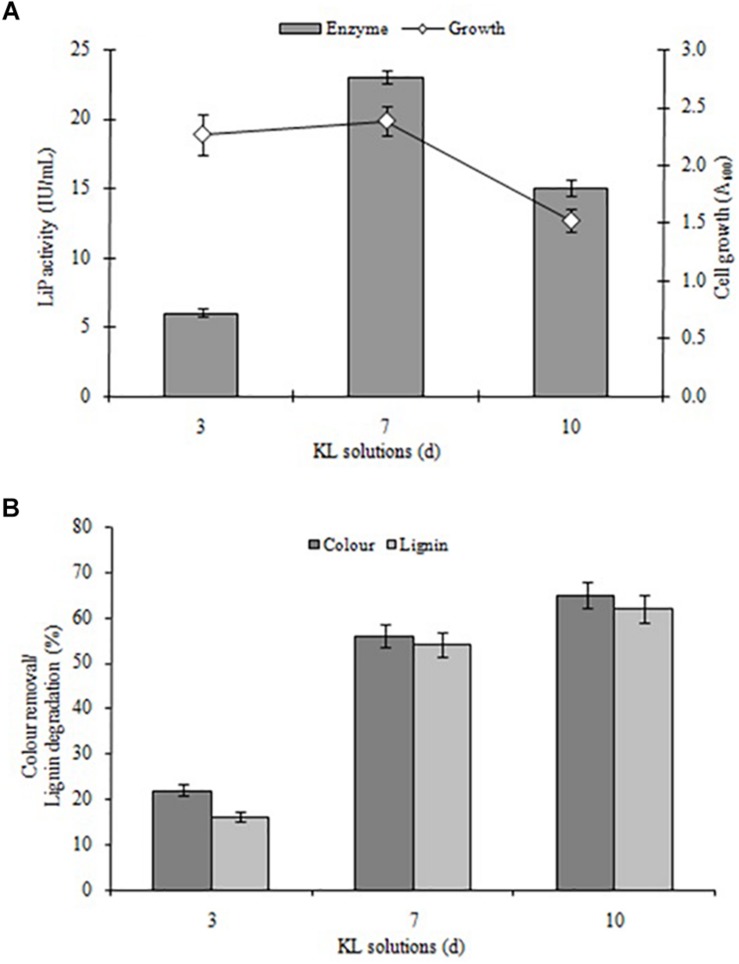
**(A)** Bacterial growth and LiP activity and **(B)** KL degradation and decolorization induced by *S. liquefaciens* at different time points (72, 168, and 240 h, equivalent to 3, 7, and 10 days). The study was conducted at 30 ± 1°C with shaking at 120 rpm. Values are mean ± *SD* (*n* = 3).

Kraft lignin degradation conducted and confirmed by LiP-producing *S. liquefaciens*, previously this strain has shown lignin degradation and color removal of paper mill effluents (Haq et al., 2016). The KL degradation and decolorization experiments performed for quantifying the degradation rate of KL and color removal, and examined in specific time intervals (0, 72, 168, and 240 h) in MSM broth (pH 7.6) containing 1000 mg L^–1^ KL. The KL degradation was spectrophotometrically characterized at different time periods. The results with respect to bacterial growth, LiP activity, KL decolorization, and KL degradation indicated that growth and LiP activity (23 IU/ml) peaked at 168 h and then declined, whereas the maximum KL degradation as 65% and 62% decolorization were observed in 240 h (Figures 2A,B). The COD reduction during bacterial treatment was also measured and it was recorded that initial COD of 4030 mg/l was decreased to 1289 mg/l (68%) after 240 h of treatment (data not shown). The above results are quite improved in contrast to a recent study on KL decolorization and degradation induced by *Pandoraea* sp. ISTKB reported 41% degradation and 36% decolorization after 48 h (Kumar et al., 2015). The KL degradation rate 65% of *S. liquefaciens* is quite higher than reported for *Bacillus* sp. ITRC S8 (37%), *A. aneurinilyticus* ITRC S7 (33%), and *Paenibacillus* sp. ITRC S6 (30%) (Chandra et al., 2007). These findings indicate that bacteria are able to grow and produce improved LiP enzyme to induce KL degradation and its biotransformation.

Furthermore, it was observed that the KL content of MSM broth decreased during the initial hours of incubation despite the rapid bacterial growth. This lower decolorization in the initial phase (despite fast bacterial growth) might be due to the use of simple carbon (glucose) and peptone sources available from MSM growth media were only compulsory to use KL as a co-substrate after the depletion of these sources. An earlier study on the degradation and decolorization of KL (500 mg L^–1^) reported 30–40 and 40–65% degradation due to *Paenibacillus* and *Bacillus*, respectively, with requirements for additional carbon and nitrogen sources (Chandra et al., 2007). The findings of this research are also consistent with studies by Ahmad et al. (2010) and Sainsbury et al. (2013).

### KL Surface Characterization After Treatment by *Serratia liquefaciens*

**FIGURE 3:**
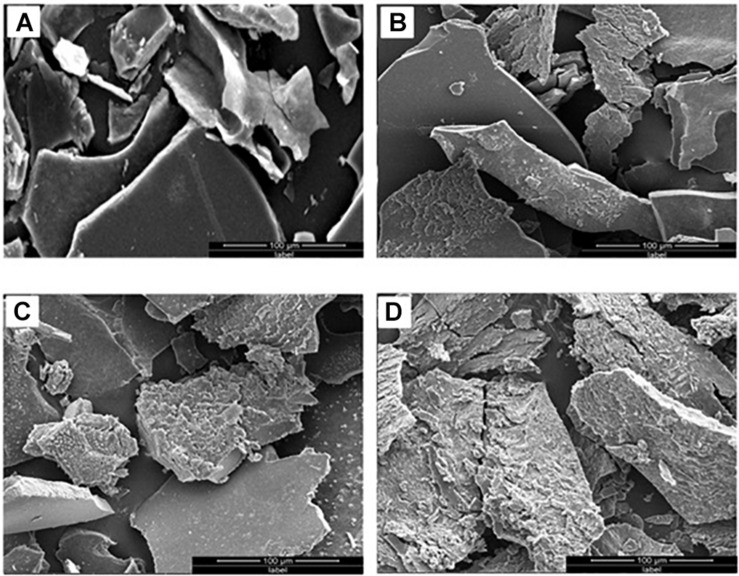
SEM images of **(A)** untreated KL and bacterial-treated KL solution after **(B)** 72, **(C)** 168, and **(D)** 240 h of incubation.

The KL structural transformation process was studied by SEM analysis at different times. The magnification and scaling were kept similar in all SEM images. The SEM image of the KL isolated from the control (untreated) solution revealed that the rounded shapes of the KL particles became flat, with smooth surfaces, after dissolving in assay medium (MSM broth + bacteria) as shown in Figure 3A. The SEM images of the bacteria-degraded KL showed that the smooth surfaces eroded, which increased with incubation time shown in Figures 3B–D. This change was due to bacterial degradation, and the degradation was maximum observed after 240 h of incubation. Thus, the SEM images indicate significant degradation/transformation of KL structurally by *S. liquefaciens*. There has been a comparable finding reported by researchers who reported substantial degradation/biotransformation of KL by *Pandoraea* sp. ISTKB (Kumar et al., 2015). In a recent study it is found that *S. liquefaciens-*biotransformed alkali lignin exhibited less phototoxicity and cytotoxicity than untreated alkali lignin (Rybczyńska-Tkaczyk and Korniłłowicz-Kowalska, 2017).

**FIGURE 4:**
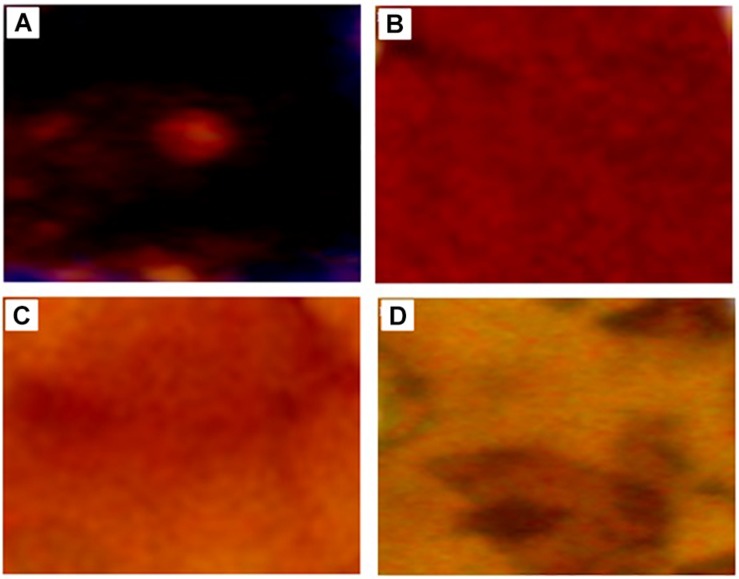
Stereoscope microscopy images of **(A)** untreated KL and bacterial-treated KL solution after **(B)** 72, **(C)** 168, and **(D)** 240 h of incubation.

Morphological changes of KL during the degradation process were also evaluated by stereo microscopy shown in Figure 4, which revealed the dark color of the untreated KL. However, with degradation, the dark color was gradually reduced due to the erosion of the KL surface. This indicates that lignin degradation occurs throughout constantly degradation process.

### KL Characterization by DLS

Dynamic light scattering experiments to assess the *D*_H_ of the untreated and bacteria-treated KL samples were also performed. The peak for the untreated KL sample corresponds to a *D*_H_ of 334.8 nm. After treatment by bacteria, the overall size of KL particles was decreased compared to the control of KL particles. After 72, 168, and 240 h, the KL size decreased by 10.2, 12.6, and 18.0%, with corresponding *D*_H_ values of 300.4, 292.8, and 275.1 nm, respectively, data shown in Table 1. The results suggested that after bacterial treatment, the larger KL particles became smaller compared to the control of KL particles. Similar results were recently described after Lignosulfonate degradation induced by *Sphingobacterium* sp. HY-H and KL degradation induced by *Penicillium chrysogenum var. halophenolicum* (Wang et al., 2013; Remedios et al., 2016).

**TABLE 1:** Mean particles size (hydrodynamic diameter, *D*_H_) of control and bacterial-treated KL samples estimated by dynamic light scattering (DLS).

**Sample**	**Peak (nm)**
UT	334.8
BT (72 h)	300.4
BT (168 h)	292.8
BT (240 h)	275.1

*UT, untreated; BT, bacteria-treated.*

### KL Characterization by ATR–FTIR

**FIGURE 5:**
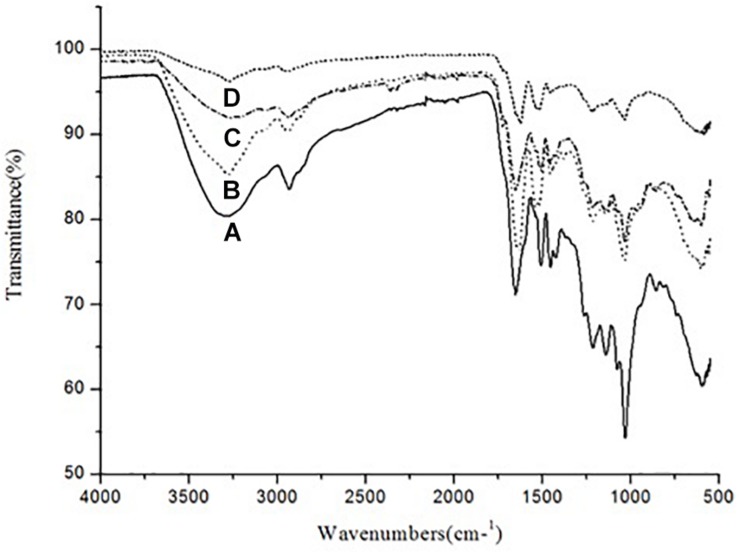
ATR–FTIR spectra of **(A)** untreated KL and bacterial-treated KL solution after **(B)** 72, **(C)** 168, and **(D)** 240 h of incubation.

The ATR–FTIR analysis was carried out in order to analyze the changes in functional group composition of KL after bacterial treatment, on the untreated and bacterial-treated sample. There were differences between the ATR–FTIR spectra for the control sample and the bacterial-treated samples at various time points (72, 168, and 240 h), as shown in Figure 5.

The control sample had absorption bands at around 3289 and 2932 cm^–1^. The band at 3289 cm^–1^ was replaced by bands at 3274, 3275, and 3269 cm^–1^ after 72, 168, and 240 h of bacterial treatment, respectively. Similarly, the band at 2932 cm^–1^ was replaced by bands at 2934, 2935, and 2934 cm^–1^ after bacterial treatment, respectively. Thus, O–H, phenolic, aromatic, methyl, and methoxy groups were likely rearranged and transformed by *S. liquefaciens* to form new metabolites. The most prominent bands at around 1600 cm^–1^ assigned to aromatic skeletal vibrations were 1651, 1626, and 1627 cm^–1^ for O–H and conjugated C–O stretching. The peaks in the bacterial treated samples decreased in a time-dependent stepwise manner. These results were caused by changes in the number of carbonyl groups substituted at the para position of phenyl rings. The peaks at around 1500 cm^–1^ were attributable to KL, as the bands arise purely due to aromatic skeletal vibration (C-C) in the KL. The peaks at around 1450 cm^–1^ were triggered by the extending of the KL phenol-ethers (Xu et al., 2013), while the 1210 cm^–1^ peak represents C–O stretching and C–O linkage in guaiacyl aromatic methoxyl groups, and the 1140 cm^–1^ peak represents syringyl ring and C–O stretching in the KL (Pandey and Pitman, 2003). The bands at 1038 cm^–1^ were due to guaiacyl (G) and syringyl (S) groups. The analysis of the ATR–FTIR spectra of the bacterial-treated KL samples evidenced the occurrence of delignification in a time-dependent manner.

### KL Characterization by GC–MS/MS Analysis

**FIGURE 6:**
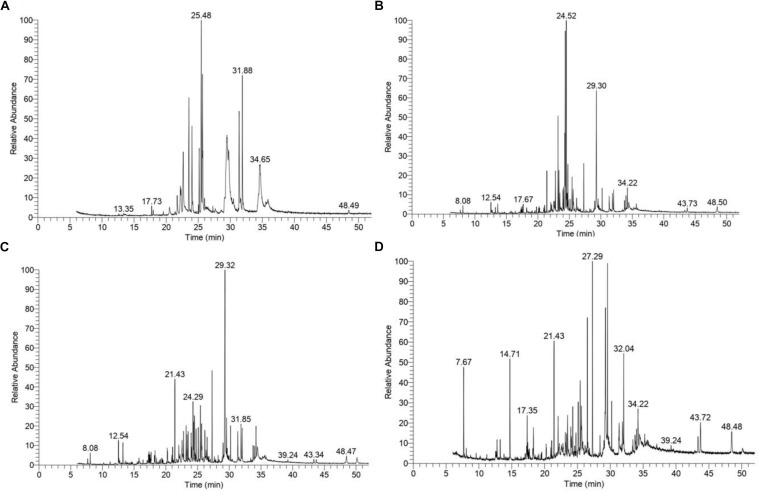
Gas chromatography–mass spectrometry(GC–MS) chromatograms of compounds extracted with ethyl acetate from **(A)** untreated KL and bacteria-treated KL solution after **(B)** 72, **(C)** 168, and **(D)** 240 h of incubation. The MS-identified compounds with respect to their retention times are given in Table 2.

The GC–MS assessment has further verified KL’s bacterial biotransformation. The result has highlighted in Figures 6A–D and Table 2. GC–MS of ethyl extracts of untreated KL solution (Figure 6A) has shown the presence of various organic compounds such as hexadecanoic acid (RT = 27.29), 1,5-pentanediyl ester (RT = 13.35), 2-allyl-5-*t*-butylhydroquinone (RT = 17.73), dl-alanyl-l-leucine (RT = 22.22), 1,4-diaza-2,5-dioxobicyclo nonane (RT = 22.66), 5-chlorobenzimidazole-2-carboxylic acid (RT = 23.55), 2-non-enoic acid (RT = 23.76), 5-chlorobenzimidazole-2-carboxylic acid (24.05), 1,4-diaza-2,5-dioxo-3-isobutyl bicycle nonane (RT = 25.48), 2,5-piperazinedione, 3,6-*bis*(2-methylpropyl) (RT = 29.48), 3-benzyl-1,4-diaza-2,5-dioxobicyclo nonane (RT = 31.88), 2-acetylamino-3-phenylpropionic acid, 1-carbamoylethyl ester (RT = 34.65), and *N*,*N*′-dicyclohexyl-1-cyano-7-pyrrolidinylperylene-3,4:9,10-tetracarboxylic acid bisimide (RT = 48.47). However, these compounds were not diminished in the ethyl acetate extract of the bacterial treated KL solution (Figures 6B–D).

**TABLE 2:** Compound identified as trimethylsilyl (TMS) derivatives in ethyl extract from untreated (<24 h) and bacterial-treated (72, 168, and 240 h) KL samples as given in Figure 6.

**Retention time (min)**	**Compound**	**Present/absent (h)**			
		**<24**	**72**	**168**	**240**
7.67	Propanoic acid	−	−	−	+
12.54	2-Methyl-2,3-dihydro-1H-benz[g] indole	−	+	+	−
13.35	Hexadecanoic acid, 1,5-pentanediyl ester	+	−	−	−
14.71	2-Butenoic acid	−	−	−	+
17.35	Docosane	−	−	−	+
17.67	Glycine	−	+	−	−
17.73	2-Allyl-5-*t*-butylhydroquinone	+	−	−	−
21.43	Methyl 1-methyl-2-oxo-*trans*-4-[*trans*-4-(*trans*-4-propylcyclohexyl)cyclohexyl]-r-1-cyclohexane carboxylate	−	+	+	+
22.22	dl-Alanyl-l-leucine	+	−	−	−
22.66	1,4-Diaza-2,5-dioxobicyclo nonane	+	−	−	−
22.79	D-Ribo-hexonic acid	−	+	−	−
23.34	D-Fructose	−	+	−	−
23.55	5-Chlorobenzimidazole-2-carboxylic acid	+	−	−	−
23.76	2-Non-enoic acid	+	−	−	−
24.03	5-Chlorobenzimidazole-2-carboxylic acid	+	−	−	−
24.52	D-Fructose	−	+	−	−
25.48	1,4-Diaza-2,5-dioxo-3-isobutyl bicycle nonane	+	−	−	−
26.36	3,4-Diethynyl-1,6-*bis*[(triisopropylsilyl)ethynyl]hex-3-ene-1,5-diyne	−	+	−	−
27.29	Hexadecanoic acid	−	−	+	+
29.30	*t*-Butyl 2-[1′-cyano-1′-(2″-cyanophenyl)methyl]-2-methyl thio-3,4,5,6-tetrahydro-1,3-thiazine-3-carboxylate	−	+	−	−
29.32	1,4-Diethynyl-2,5-dipropoxybenzene	−	−	+	−
29.48	2,5-Piperazinedione, 3,6-*bis*(2-methylpropyl)	+	−	−	−
31.85	Pyrrolo[1,2-a]pyrazine-1,4-dione, hexahydro-3-(phenylmethyl)-	−	−	+	−
31.88	3-Benzyl-1,4-diaza-2,5-dioxobicyclo nonane	+	−	−	−
32.04	*n*-Tetracosanol-1	−	−	−	+
32.22	Thiopheno[b,b′]dicamphore 1,1-dioxide	−	−	−	+
34.22	2-Methoxy-6-(trimethylsilyl)benzo[a]fluorene-11	−	+	−	−
34.65	2-Acetylamino-3-phenylpropionic acid, 1-carbamoylethyl ester	+	−	−	−
39.24	Cholesterol	−	−	+	+
43.34	1-Heptatriacotanol	−	−	+	−
43.72	1,8-Diphenyl-3,4,10,11-tetrahydro[1,4]dioxino[2,3-g:5,6-g′]diisoquinoline methyl	−	−	−	+
43.73	3,5,7-*Tris*(trimethylsiloxy)-2-[3,4-di(trimethylsiloxy)phenyl]-4H-1-benzopyran-4-one	−	+	−	−
48.47	*N*,*N*′-Dicyclohexyl-1-cyano-7-pyrrolidinylperylene-3,4:9,10-tetracarboxylic acid bisimide	+	−	+	+

Further, the analysis of the bacterial treated KL solutions indicated the presence of several new peaks at various time points (Figures 6B–D). At the end of the experiment (240 h), the new peaks detected as metabolites were: propanoic acid (RT = 7.67), 2-butenoic acid (RT = 14.71), docosane (RT = 17.35), *N*-tetracosanol-1 (RT = 32.04), thiopheno[b,b′] dicamphore 1,1-dioxide (RT = 32.22), and 1,8-diphenyl-3,4,10,11-tetrahydro [1,4]dioxino[2,3-g:5,6-g′]diisoquinolinemethyl (RT = 43.72). New compounds such as 2-methyl-2,3-dihydro-1H-benz[g] indole (RT = 12.54) were found at both 72 and 240 h, while hexadecanoic acid (RT = 27.29), cholesterol (RT = 39.24), *N*,*N*′-dicyclohexyl-1-cyano-7-pyrrolidinylperylene-3,4:9,10-tetracarboxylic acid bisimide (RT = 48.47) were found at both 168 and 240 h. Methyl 1-methyl-2-oxo-*trans*-4-[*trans*-4-(*trans*-4-propylcyclohexyl) cyclohexyl]-*r*-1-cyclohexanecarboxylate (RT = 21.43) was detected in all degraded samples. The results regarding the disappearance of compounds identified in the control sample and the formation of new peaks in the analysis of the degraded samples suggested that the bacterial strain has a strong ability to transform KL through metabolic activity. Most of the compounds detected were reported earlier during the bacterial degradation of KL and paper mill effluent (Raj et al., 2007; Abhishek et al., 2017).

### Cytotoxicity Evaluation of KL

**FIGURE 7:**
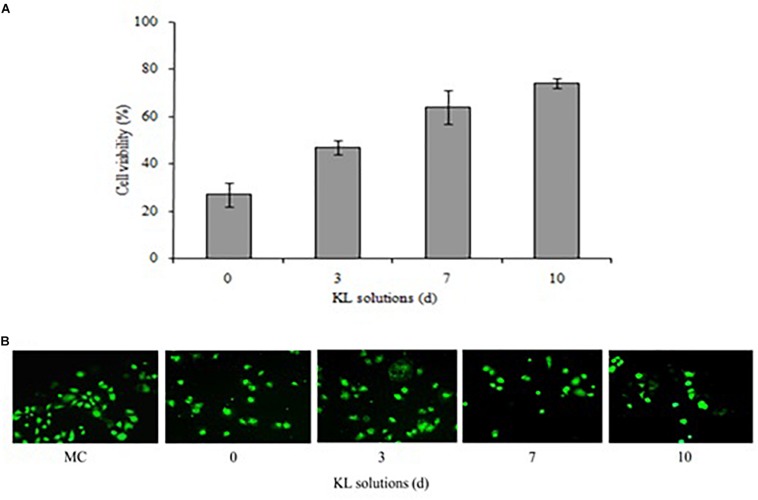
**(A,B)** Cytotoxicity of untreated (<24 h) and bacterial-treated (72, 168, and 240 h) KL solution extracts using the NRK-52E cell line, as assessed by **(A)** MTT assay and **(B)** calcein-AM assay after exposure for 24 h. MC, medium control. Values are mean ± *SD* (*n* = 3).

The cytotoxicity potential of the different KL solutions was determined using MTT and calcein-AM assays with a human kidney cell line (NRK-52E). In the MTT assay, after 24 h of exposure to the untreated (<24 h) and bacterial treated (72, 168, and 240 h) KL solutions, the cell survival rate was 27, 47, 64, and 74%, respectively. This indicated an increase in cell survival after bacterial treatment shown in Figure 7A. However, the MTT assay indicated that both untreated and bacterial treated KL solutions were cytotoxic to human kidney cells. Previous studies have reported on the cytotoxicity of paper mill effluents, based on MTT assays using various human cell lines (Abhishek et al., 2017). Further, the calcein-AM assay showed that, while the cell viability of the media control (MC) was unaffected by calcein-AM staining, a gradual increase in the cell viability was observed as the KL degradation increased shown in Figure 7B. Thus, after bacterial treatment of the KL solutions, cell proliferation and viability increased. The result suggested that the cytotoxicity of bacterial-treated KL solutions decreased significantly but not completely. All know it that there is no standard available for the lignin. Lignins are different types and their stricture is depending on the isolation source and methods. Hence, it is not possible to compare the results of the present work conducted with KL with those that were published previously. However, various studies reported cytotoxicity of lignins on various cell lines (Gordobil et al., 2019). Biodegradation along with detoxification reveals the potential of the bacterium to be used for lignin degradation (Barapatre et al., 2016).

## Conclusion

Kraft lignin along with other lignins derivate compounds are the chief ingredients of paper mill effluents, which contribute to a dark appearance in color and pollution to water bodies. In the present study, we reported biodegradation/biotransformation KL by ligninolytic *S. liquefaciens* at very high concentration (1000 mg L^–1^) with significant reduction of KL (65%) and color (62%). The KL biodegradation process of isolate was verified by instrumental analysis using SEM, DLS, ATR–FTIR, and GC–MS. The KL was transformed into various less toxic metabolites. Further, bacterial treatment of KL led significant reduction in cytotoxicity. The above study can be concluded, S. *liquefaciens* could be an important tool to degrade KL by transforming it into various less toxic compounds as shown above by transforming and minimizing of cytotoxicity effects on kidney cell lines. Hence, concern bacteria may be useful to keep the environment free and safe from KL and lignin-derived pollutants.

## Data Availability Statement

The raw data supporting the conclusions of this manuscript will be made available by the authors, without undue reservation, to any qualified researcher.

## Author Contributions

AS was the first presenting author, and performed the lignin biodegradation, biotransformation assay, cytotoxicity assay, and GC–MS/MS extraction analysis. PY prepared the tables of manuscript and performed the sample preparation for GC–MS/MS, ATR–FTIR, DLS, SEM, and EDX analyses. RB performed the statistical analysis and the interpretation of the results. GS performed and provided the input in statistical tests, figures, and overall manuscript editing with some corrections. AR provided the intellectual input, designed the study, and approved the protocols which have been followed in the study and finally carried out manuscript correction, proofread, and communicated the same.

## Conflict of Interest

The authors declare that the research was conducted in the absence of any commercial or financial relationships that could be construed as a potential conflict of interest.
